# Improved reinforcement learning path planning algorithm integrating prior knowledge

**DOI:** 10.1371/journal.pone.0284942

**Published:** 2023-05-04

**Authors:** Zhen Shi, Keyin Wang, Jianhui Zhang

**Affiliations:** 1 School of Automotive Engineering, Hubei University of Automotive Technology, Shiyan, Hubei, China; 2 Key Laboratory of Automotive Power Train and Electronics (Hubei University of Automotive Technology), Shiyan, Hubei, China; Zhejiang University of Technology, CHINA

## Abstract

In order to realize the optimization of autonomous navigation of mobile robot under the condition of partial environmental knowledge known. An improved Q-learning reinforcement learning algorithm based on prior knowledge is proposed to solve the problem of slow convergence and low learning efficiency in mobile robot path planning. Prior knowledge is used to initialize the Q-value, so as to guide the agent to move toward the target direction with a greater probability from the early stage of the algorithm, eliminating a large number of invalid iterations. The greedy factor ε is dynamically adjusted based on the number of times the agent successfully reaches the target position, so as to better balance exploration and exploitation and accelerate convergence. Simulation results show that the improved Q-learning algorithm has a faster convergence rate and higher learning efficiency than the traditional algorithm. The improved algorithm has practical significance for improving the efficiency of autonomous navigation of mobile robots.

## 1. Introduction

In recent years, with the popularity of artificial intelligence, mobile robot technology has also developed rapidly, and its application fields have become more and more extensive. How to realize the autonomous navigation of mobile robot in a given environment is the key to realize its function [1–3]. An important part of mobile robot research is path planning, i.e., searching for an optimal or nearly optimal collision-free path from the initial state to the target state according to a certain performance index [4]. Path planning can be either global or local. Global path planning depends on a robot’s grasp of global knowledge in the operating environment. Global path planning algorithms include the A* algorithm [5–7], visibility graph [8–10], cell decomposition [11] and Dijkstra algorithm [12]. Local path planning depends on real-time information from sensors. Typical algorithms include the artificial potential field method [13], genetic algorithm [14], neural network method [15], and reinforcement learning (RL) algorithm [16]. RL has received much attention because it can find the optimal path through trial and error in an unknown environment, RL is applied in some industrial fields, such as flow-shop scheduling [11, 17].

Q-learning is one of the most widely and successfully applied RL algorithms in mobile robot path planning [18, 19], but it faces two challenges: (1) the RL agent searches blindly in the early stage of the algorithm, resulting in too many invalid iterations; and (2) it is difficult to balance exploration and exploitation. Wen et al. [20] proposed a method of initializing the Q-value based on fuzzy logic in the early stage, so the agent no longer blindly selects an action, which decreases invalid iterations and speeds up the algorithm. Viet et al. solved the path planning problem with the Dyna-Q-learning algorithm, which combines the Dyna learning framework and Q-learning algorithm. Both of the above approaches increase the complexity of the algorithm.

This paper proposes an improved Q-learning algorithm that: (1) combines the prior knowledge to initialize the Q-value, where the closer to the target location the larger the Q-value, so that the agent can search toward the target location in the early stage, eliminating a large number of invalid iterations; and (2) dynamically adjusts the greedy factor ε according to the number of successful arrivals at the target location. In the early stage of the algorithm, ε is large and the agent tends to explore the environment, then ε is gradually decreased, and the agent tends to make use of high-quality actions, so as to better balance exploration and exploitation. It is expected that the improved algorithm can solve the shortcomings of the general Q-learning algorithm applied to the path planning of mobile robots, such as slow convergence speed, long iteration time, and difficulty in balancing the relationship between exploration and utilization.

The rest of this paper is organized as follows. RL and traditional Q-learning are introduced in section II. Section III proposes improvements to traditional Q-learning. Section IV presents experimental results and analysis, and section V presents our conclusions.

## 2. Related work

### 2.1 Reinforcement learning

**Fig 1:**
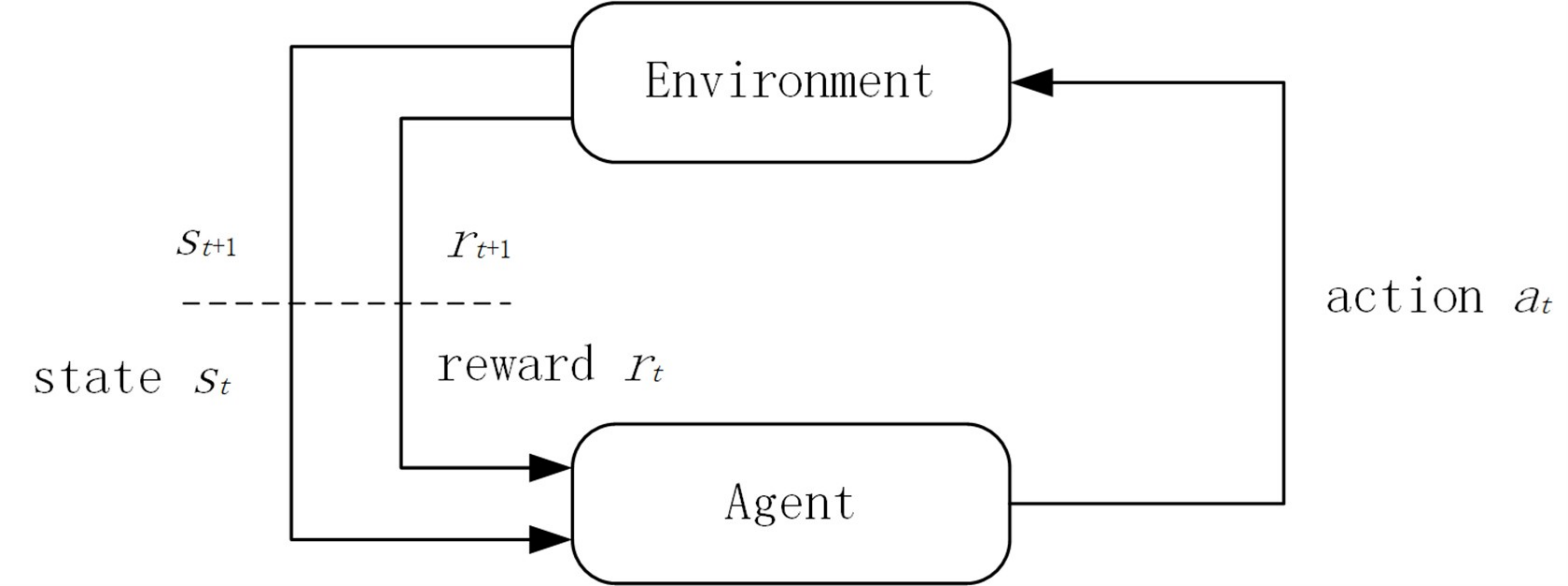
RL model.

RL is different from supervised and unsupervised learning. It realizes the optimal solution of sequential decision-making problems through an agent interacting with the environment. An RL problem can be represented by a Markov decision process [21], which is defined as a 5-tuple (S, A, P, R, γ), where S is a set of states, A is a set of actions, P is the state transition probability, R is a reward function, and γ is a discount factor. The process of RL is illustrated in the diagram in Fig 1.

An agent selects action at in A to interact with an environment, which transitions from state st to state st+1 and returns a reward rt+1 to the agent. RL aims to determine the optimal strategy to maximize the cumulative reward in a given environment. For a given policy, the cumulative reward Gt is defined to quantify the value of the state:

$$G_{t}=r_{t+1}+{\gamma r}_{t+2}+\gamma^{2}r_{t+3}+\ldots =\sum_{k=0}^{\infty }\gamma^{k}r_{t+k+1}$$ (1)

where γ∈[0,1] is a variable that determines the way future rewards are valued. A larger γ means that future rewards are more important.

To evaluate the state and action, the state value function V(s) and state-action value function Q(s, a) are introduced as follows:

$$V(s_{t})=E[\sum_{k=0}^{\infty }\gamma^{k}r_{t+k+1}|s_{t}],$$ (2)

$$Q(s_{t},a_{t})=E[\sum_{k=0}^{\infty }\gamma^{k}r_{t+k+1}|s_{t},a_{t}],$$ (3)

Since both states and actions occur randomly with a certain probability, the above two equations are expressed by the expected values. Their Bellman equations are as follows:

$$V(s_{t})=E[r_{t+1}+\gamma V(s_{t+1})|s_{t}],$$ (4)

$$Q(s_{t},a_{t})=E[r_{t+1}+\gamma Q(s_{t+1},a_{t+1})|s_{t},a_{t}],$$ (5)

The optimal state value function and optimal state-action value functions can be respectively expressed as:

$$V^{*}(s_{t})=\underset{\pi }{\max}V^{\pi }(s_{t}),$$ (6)

$$Q^{*}(s_{t},a_{t})=\underset{\pi }{\max}Q^{\pi }(s_{t},a_{t}),$$ (7)

where π is the policy.

Q*(*s*_*t*_,*a*_*t*_) is the cumulative reward corresponding to the optimal action in the optimal policy. Therefore,

$$Q^{*}(s_{t},a_{t})>V^{*}(s_{t}),$$ (8)

and the optimization problem of reinforcement learning can be expressed as

$$\pi^{*}(a_{t}|s_{t})=\underset{\pi }{\arg\;\max}Q^{*}(s_{t},a_{t}),$$ (9)

where π*(*a*_*t*_|*s*_*t*_) is the optimal policy for the RL agent.

### 2.2 Q-learning

Q-learning is an offline temporal difference RL algorithm [15] that follows a different policy when selecting an action than when updating it. To speed up the update process of the state-action value function, Q-learning directly selects the maximum state-action value function corresponding to the next state to participate in the update, expressed as

$$Q(s_{t},a_{t})\leftarrow Q(s_{t},a_{t})+\alpha [r_{t+1}+\gamma \underset{a}{\max}Q(s_{t+1},a)-Q(s_{t},a_{t})],$$ (10)

where α∈(0,1) is the learning rate.

The Q-learning algorithm is given as follows:

1. Set the discount factor *γ*, learning rate α, and reward *r*;
2. Initialize matrix *Q*;
3. For each episode:
4. Select an initial state *s*;
5. While the goal state has not been reached:
6. Select an action *a* in action set *A* based on a policy;
7. Perform *a* and reach the next state *s’* to obtain reward *r*;
8. Update matrix *Q*;
9. Set *s’* as *s*;
10. Until *s* is the goal state.

## 3. Improved Q-learning path planning algorithm

### 3.1 Initialization of Q-value function

In the traditional Q-learning algorithm, the Q-value is initialized to 0 or a random number, which leads to blind selection at the early stage, resulting in a large number of invalid iterations. For this problem, we combine the prior knowledge to initialize the Q-value. This study aims to investigate the situation in which the initial position and target position of the mobile robot are known, and the position of the obstacle is unknown. We use this known environmental information to initialize the Q-value.

In a grid map, each grid represents a state. The state value V(s’) of each state in the environment is obtained as

$$V(s’)=\zeta *(1-\frac{\rho (s’)}{\rho_{\max}}),$$ (11)

where ζ is a positive coefficient, ρ(*s*’) is the distance from state s to the target position, and ρ_max_ is the diagonal length of the grid map.

Then the Q-value is initialized according to the relationship between the state action value and state value as follows:

$$Q(s,a)=r+\gamma \sum_{s’}P(s’|s,a)V(s’),$$ (12)

where P(s’|s,a) is the probability of transferring to state s’ when the current state s and action a are determined.

According to this method, the Q-value is initialized, and the known environmental information is fully used, so that the closer the target the larger the Q-value. In the early stage, the agent will move toward the target with greater probability, decreasing the number of invalid iterations and accelerating convergence.

### 3.2 Dynamic adjustment of greedy factor ε

In traditional Q-learning, the ε-greedy policy is applied to select an action. The agent explores the environment with probability ε, and exploits the optimal action with probability 1-ε. Usually, ε takes a smaller value to ensure that better actions can somewhat balance exploration and utilization. Usually, a small ε can balance the contradiction between exploration and exploitation to a certain extent. However, when the environment is complex and the agent explores the environment with a small probability, the agent cannot be guaranteed to fully explore the environment in a limited time. To solve this problem, a dynamic adjustment strategy with greedy factor εis proposed, where ε is dynamically adjusted as

$$\varepsilon =\{\begin{array}{cc} \varepsilon_{1} & C<C1\\ \varepsilon_{2} & C1<=C<C2\\ \varepsilon_{3} & C2<=C \end{array},$$ (13)

where 0 < ε3 < ε2 < ε1 < 1, C is the number of times the agent successfully reaches the target position in the current episode, C1 and C2 are integers, and C1 < C2.

Due to the agent’s lack of environmental knowledge, early in the algorithm it has difficulty reaching the target location, and a large ε can accelerate its exploration of the environment. With the progress of the algorithm, the agent has a certain understanding of the environment, the number of times it reaches the target position increases, ε is decreased, and the exploitation of the optimal action is increased. When the algorithm reaches a certain stage and the number of times it reaches the target position exceeds a certain threshold, a small ε is set and the fast convergence of the algorithm is guaranteed.

## 4 Experiments and analysis of results

### 4.1 Experimental design

**Fig 2:**
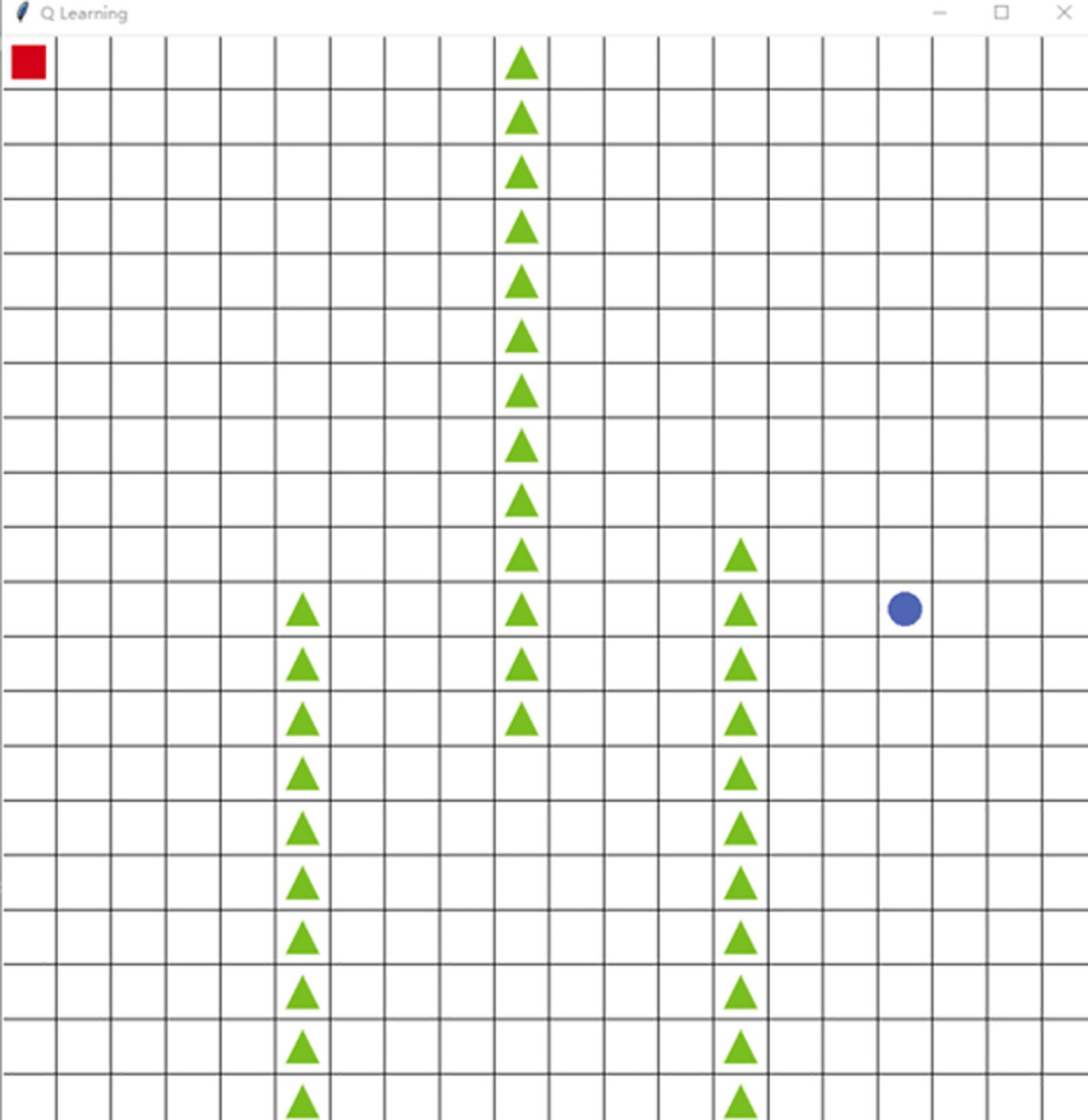
Simulation environment.

To evaluate the performance of the improved algorithm, a 20×20 grid map is built based on the Python library Tkinter, as shown in Fig 2.

The size of each grid is 20 × 20 pixels. A square represents the agent, a triangle represents an obstacle, a white grid represents a barrier-free area, the circle represents the target position, each grid represents one of 400 states, the starting point, i.e., state (1, 1), is set at position (10, 10), and the target is set at position (17, 11). The starting point and the target positions are known environmental information, and the obstacle position is unknown. When the agent encounters an obstacle or reaches the target position, an episode ends. At the end of each episode, the agent is put back at the starting position to start the next episode.

### 4.2 Experimental parameter settings

The following four algorithms are compared in the simulation environment: Q-learning (original) represents the traditional Q-learning algorithm; Q-learning (Initialization) represents an improved algorithm that integrates prior knowledge to initialize the Q value; Q-learning (Dynamic) represents the use of greedy factor dynamic adjustment strategy instead of ε-greedy strategy to improve the algorithm; Q-learning (Improved) represents the final improved algorithm proposed in this paper.

In Table 1, √ indicates that the corresponding algorithm improvement is introduced into the traditional Q-learning algorithm, and × indicates that the corresponding algorithm is not introduced.

**Table 1:** Setting of 4 kinds of algorithms.

Algorithm	Initialize Q value by fusing prior knowledge	Dynamic adjustment of ε
**Q-learning(original)**	×	×
**Q-learning(Initialization)**	√	×
**Q-learning(Dynamic)**	×	√
**Q-learning(Improved)**	√	√

The parameters of the traditional Q-learning are set as follows: ɑ = 0.01, γ = 0.9, ε = 0.2, the maximum number of episodes is 20000, and the reward function is

$$r=\{\begin{array}{cc} 1 & s’\;\mathrm{is}\;\mathrm{the}\;\mathrm{goal}\;\mathrm{position}\\ ‐1 & s’\;\mathrm{is}\;\mathrm{the}\;\mathrm{obstacle}\;\mathrm{position}\\ 0 & s’\;\mathrm{is}\;\mathrm{the}\;\mathrm{other}\;\mathrm{position} \end{array}$$ (14)

The ɑ, γ, ε, maximum number of episodes, and reward function are set to be the same in both the proposed improved Q-learning and the traditional Q-learning. Other parameters are ζ = 1, ε1 = 0.5, ε2 = 0.2, ε3 = 0.05, C1 = 1, C2 = 50.

### 4.3 Experimental results and analysis

**Fig 3:**
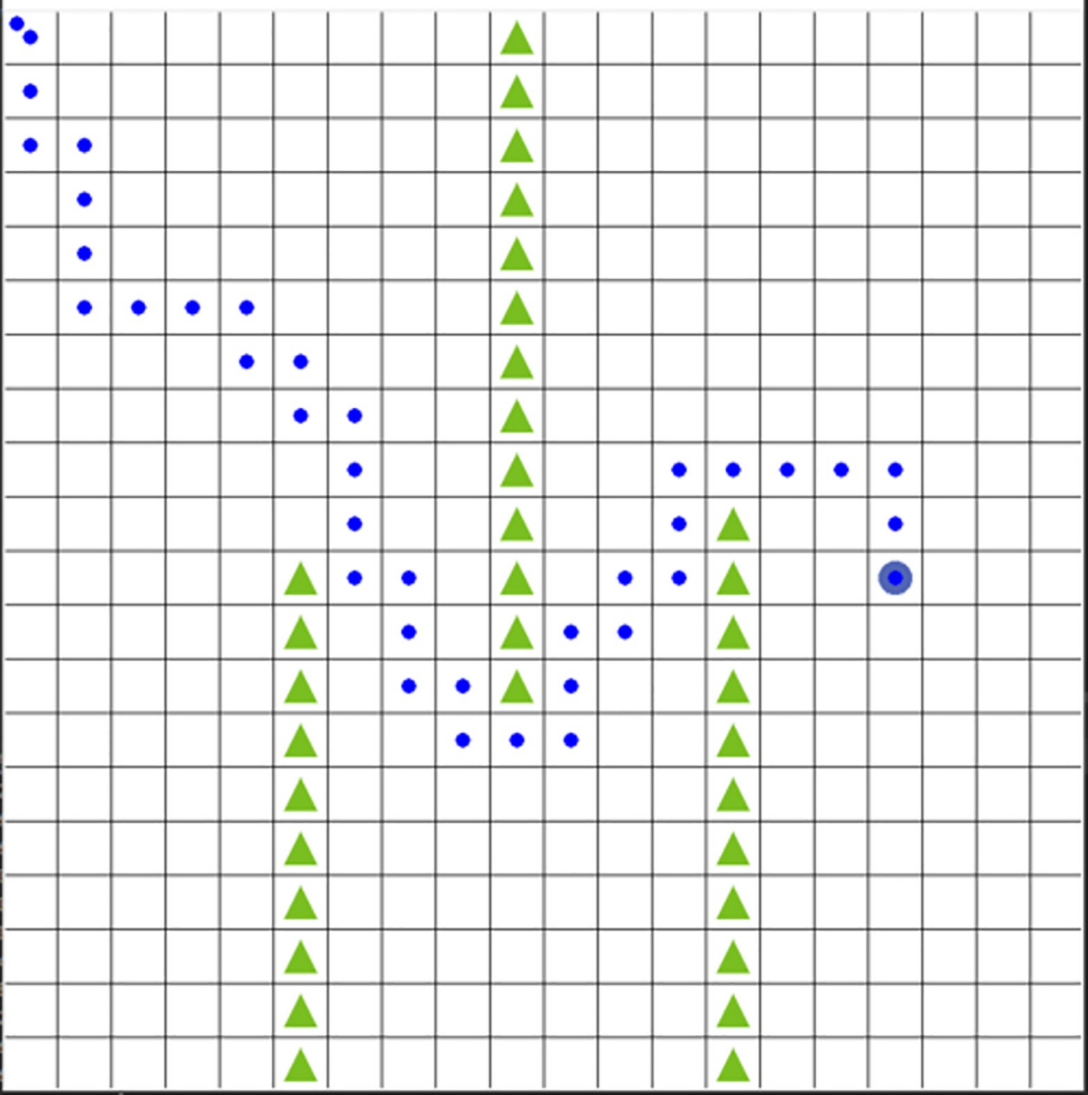
Optimal path of proposed algorithm.

The experimental results show that both algorithms can find the optimal path after a certain number of episodes. Fig 3 shows the optimal path of the proposed algorithm.

**Fig 4:**
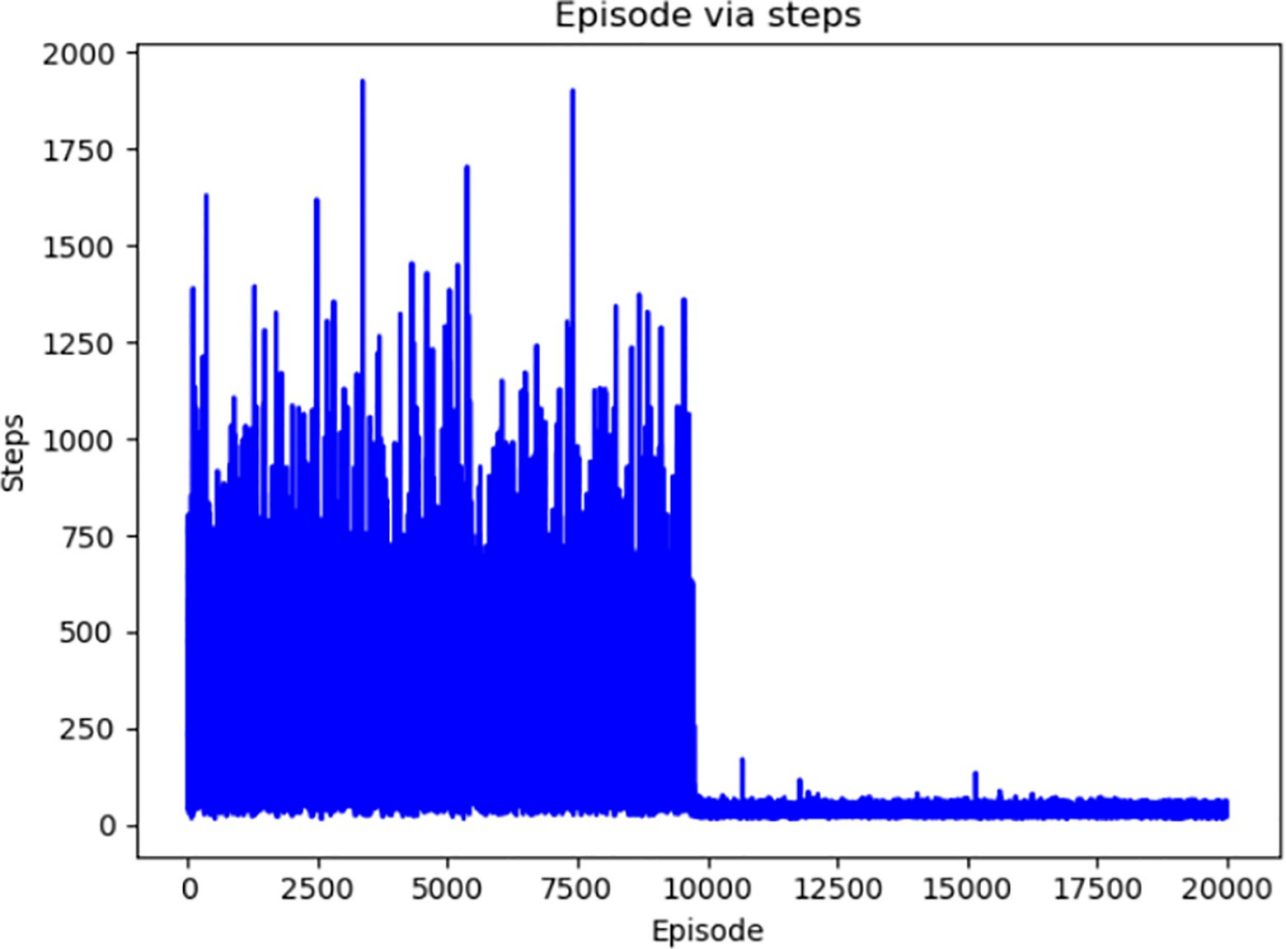
Convergence episode of traditional Q-learning.

**Fig 5:**
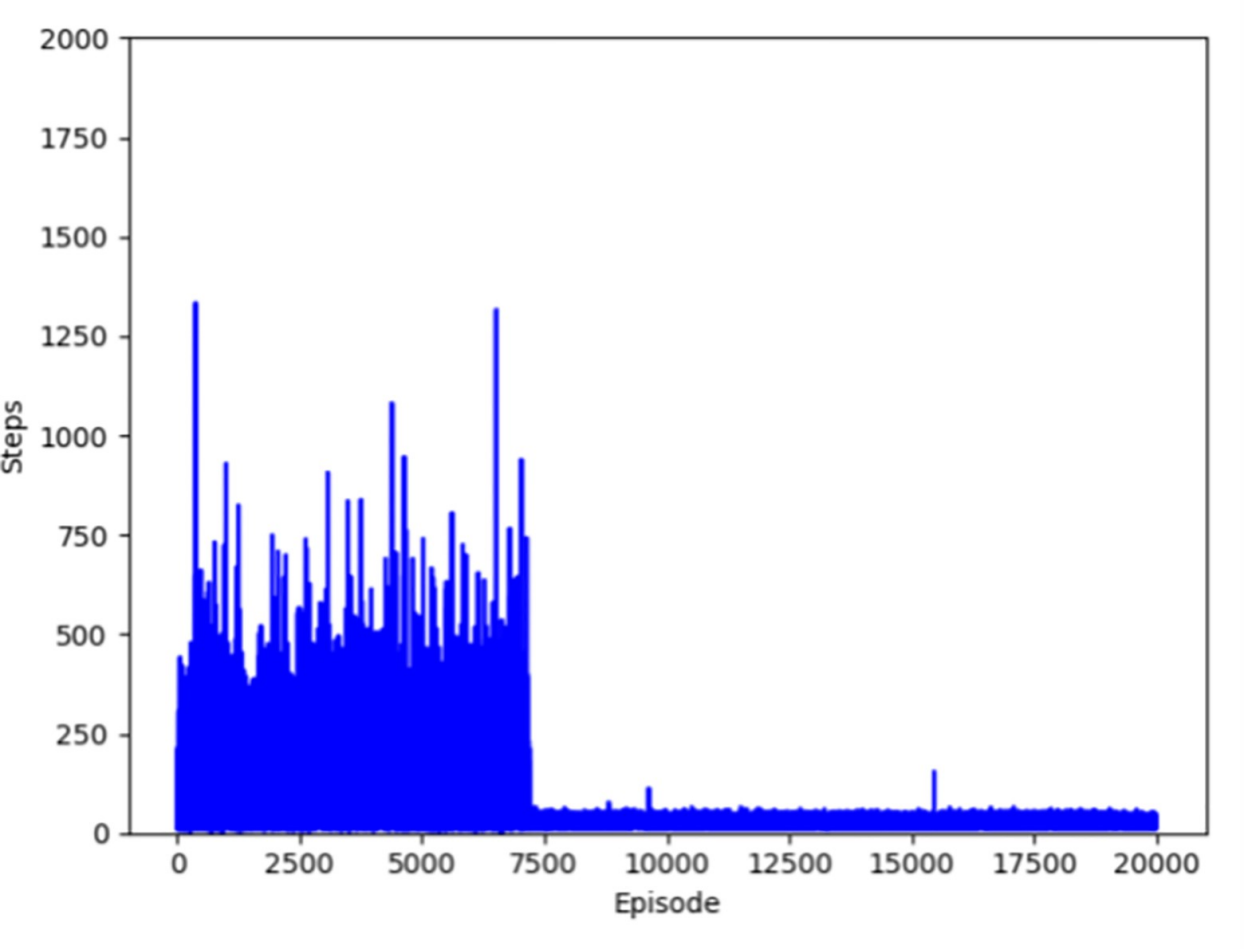
Convergence episode of Q-learning(Initialization).

**Fig 6:**
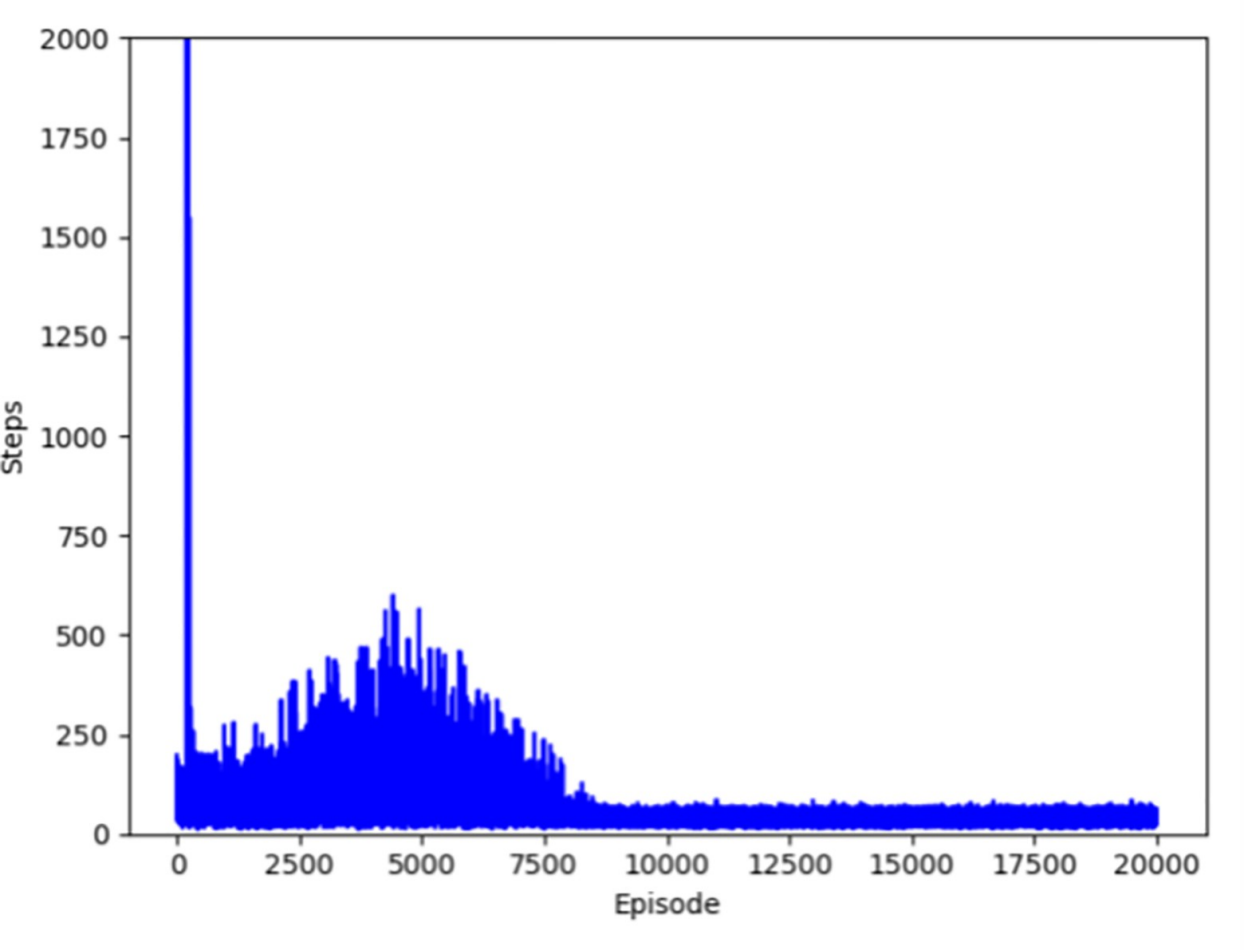
Convergence episode of Q-learning(Dynamic).

**Fig 7:**
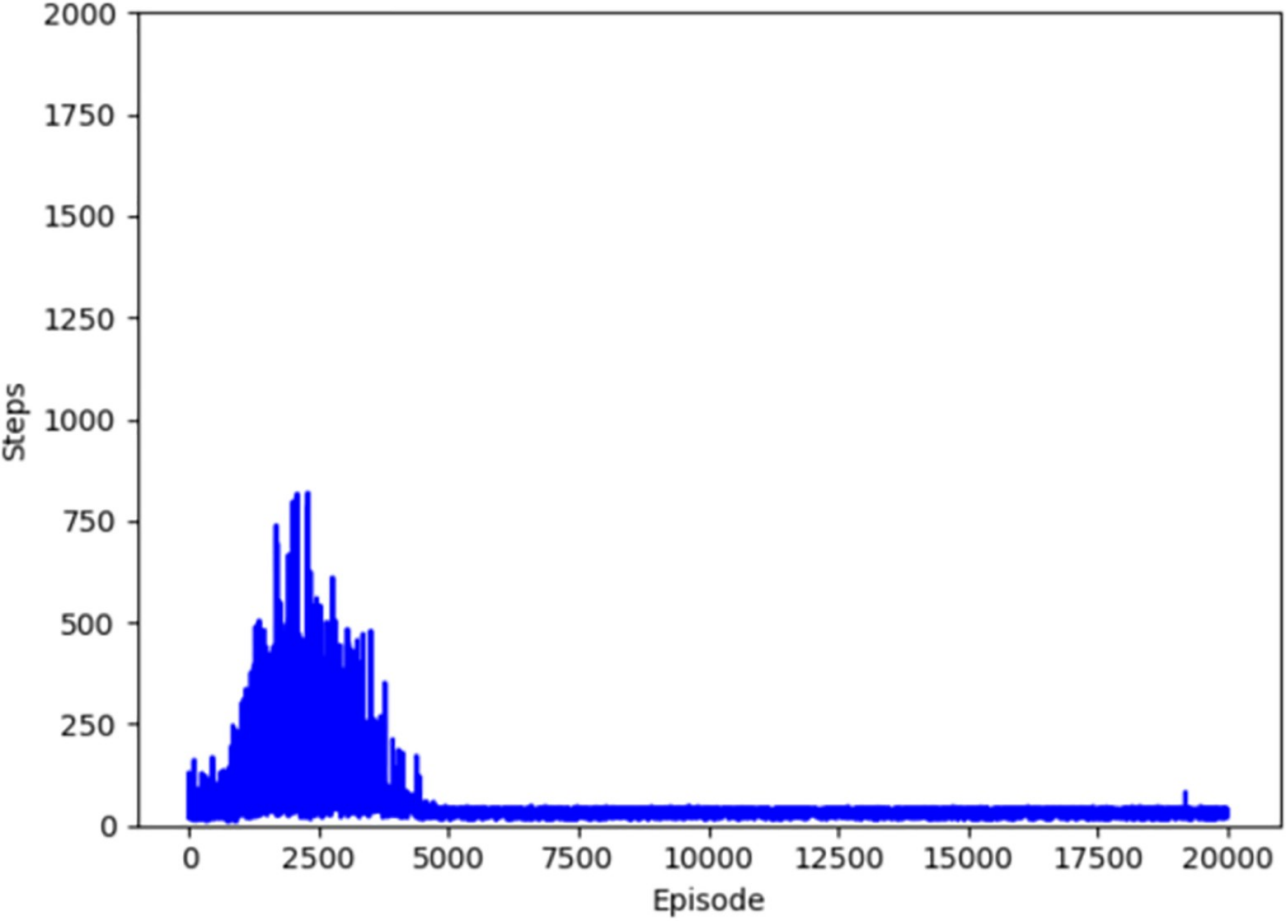
Convergence episode of improved Q-learning.

Figs 4–7 respectively show the change of steps of four algorithms mentioned above include traditional and improved Q-learning. When the number of steps fluctuates in a small range, the algorithm is considered to be convergent. Traditional Q-learning can converge in about 9500 episodes, and there are many steps in each episode before that. The improved Q-learning algorithm can converge in about 4500 episodes, with fewer running steps in each episode before convergence.

The convergence conditions are set so that that the standard deviation of the number of running steps in 10 consecutive episodes is less than 5. Each algorithm runs 20 times and takes the average value to obtain the data shown in Table 2.

**Table 2:** Performance comparison of two kinds of algorithms.

Algorithm	Run time before convergence (s)	Total running steps before convergence	Number of running episodes before convergence	optimal path (number of grids)
**Traditional Q-learning**	271.4	1747644	9387	36
**Q-learning (Initialization)**	183.6	965892	7163	36
**Q-learning (Dynamic)**	142.3	689432	5462	36
**Improved Q-learning**	127.3	462815	4631	36

By analyzing the data in Table 2, it can be seen that, compared with the traditional Q-learning algorithm, the running time before convergence of the improved Q-learning algorithm is reduced by 53.1%, the total number of steps before convergence is reduced by 73.5%, and the number of episodes before convergence is reduced by 50.7%,these data are better than the middle two algorithms. These aspects formally reflect that the improved algorithm is effective and has practical significance for robot path planning.

## 5 Conclusion

We proposed an improved Q-learning path planning algorithm. The Q-value is initialized by combining prior knowledge, which eliminates a large number of invalid iterations at the early stage of the algorithm and makes the agent move toward the target with greater probability at the beginning. The greedy factor is dynamically adjusted based on the number of times the agent successfully reaches the target position, which better balances exploration and exploitation. Simulation results based on a grid map show that the improved algorithm is more efficient and faster than the traditional algorithm. However, many parameters in the improved algorithm must be manually set according to the environment. How to more efficiently set the parameters is the focus of our next work.
